# Clinical features and treatment of 70 children with lupus anticoagulant-hypoprothrombinemia syndrome: a retrospective study from a single center in China

**DOI:** 10.1016/j.rpth.2024.102577

**Published:** 2024-09-26

**Authors:** Dandan Tian, Junfeng Zhang, Jintu Lou, Xuejun Chen, Juan Liang, Xiaojun Xu, Hui Gao, Wenjian Nie, Qing Ye, Hongqiang Shen

**Affiliations:** 1 Department of Clinical Laboratory, Children’s Hospital, Zhejiang University School of Medicine, National Clinical Research Center for Child Health, Hangzhou, China; 2 Department of Transfusion Medicine, Children’s Hospital, Zhejiang University School of Medicine, National Clinical Research Center for Child Health, Hangzhou, China; 3 Department of Hematology-Oncology, Children’s Hospital, Zhejiang University School of Medicine, National Clinical Research Center for Child Health, Hangzhou, China

**Keywords:** autoimmune diseases, children, hypoprothrombinemia, infection, lupus anticoagulant (LA)

## Abstract

**Background:**

Lupus anticoagulant-hypoprothrombinemia syndrome (LAHPS) is a rare acquired bleeding disorder characterized by the presence of lupus anticoagulant (LA) and acquired hypoprothrombinemia.

**Objectives:**

To summarize the experience of diagnosis, clinical features, and treatment of lupus anticoagulant-hypoprothrombinemia syndrome (LAHPS).

**Methods:**

A retrospective study of 70 children diagnosed with LAHPS from January 2019 to February 2024 at a single center was conducted.

**Results:**

A total of 70 subjects (32 boys and 38 girls), with a mean age of 5.58 years, were included in the study. Among these subjects, 15 had autoimmune diseases (AIDs), 51 had infections, and 4 had unknown causes. Fifty-six of 70 (80%) subjects experienced bleeding with the median bleeding score of 4, 1 of 70 (1.4%) presented with thrombosis, and 13 of 70 (18.6%) were asymptomatic. All patients exhibited prolonged prothrombin time, significantly prolonged activated partial thromboplastin time, decreased factor (F)II activity (FII:C), and positive lupus anticoagulant. There was a weak negative correlation between the severity of bleeding and FII:C level (*rs* = −0.4283; *P* < .001). Patients with infection-associated LAHPS were younger than those with AIDs-associated LAHPS (*P* < .0001). In the study, LAHPS subjects are treated with corticosteroids as the first-line therapy, or in combination with immunosuppressants. Coagulation factor replacement therapy can effectively prevent and control bleeding events. After follow-up, lupus anticoagulant of all patients had turned negative within 12 weeks. And, prothrombin time and FII:C were completely normalized of all patients without recurrence of bleeding and without thrombosis.

**Conclusion:**

Children develop LAHPS most commonly after AIDs and infection. Most patients presented with mild to moderate bleeding. The severity of bleeding symptoms was not exactly parallel to the decreased FII:C level.

## Essentials

• Lupus anticoagulant-hypoprothrombinemia syndrome (LAHPS) is a rare acquired bleeding disease.
• We analyzed the clinical data of 70 patients with LAHPS from a single center.
• Most patients with LAHPS presented with mild to moderate bleeding.
• The severity of bleeding symptoms was not exactly parallel to the decreased FII:C level.

## 1 Introduction

Lupus anticoagulant-hypoprothrombinemia syndrome (LAHPS) is a rare acquired bleeding disorder characterized by the presence of lupus anticoagulant (LA) and acquired hypoprothrombinemia caused by antiprothrombin (aPT) or antiphosphatidylserine/prothrombin (aPS/PT) complex antibodies [1]. LAHPS was first described by Rapaport et al. [1] in 1960. At present, less than 200 cases of LAHPS have been reported in the literature [2]. Previous studies on LAHPS were mainly case reports, making it difficult to establish etiology, clinical characteristics, and management, and its prevalence is uncertain. On the one hand, its rarity was attributed to clinicians who lacked experience in the diagnosis and treatment of LAHPS. On the other hand, low factor (F)VIII activity (FVIII:C) and the anti-FVIII antibody (anti-FVIII Ab) were detected in some LAHPS patients, making it difficult to distinguish those from acquired hemophilia A. Patients with LAHPS experience mild to life-threatening bleeding [3,4], so early diagnosis is crucial for LAHPS patients.

Herein, we performed a retrospective analysis of 70 subjects with LAHPS identified at a single center to explore the etiology, clinical manifestations, laboratory features, treatment, and prognosis of LAHPS. It is hoped to provide valuable reference for pediatricians in the early diagnosis and treatment of LAHPS.

## 2 Methods

### 2.1 Study population

We recruited patients from the Children’s Hospital of Zhejiang University, School of Medicine (Hangzhou, China), from January 2019 to February 2024. We determined if they fulfilled the following inclusion criteria: 1) prolonged prothrombin time (PT), 2) prolonged activated partial thromboplastin time (aPTT), 3) decreased endogenous clotting factor activity (FVIII:C, FIX:C, FXI:C, and FXII:C), 4) decreased FII activity (FII:C), 5) normal factors activity (FV:C, FVII:C, and FX:C), and 6) the presence of LA. The exclusion criteria were as follows: 1) the common causes of heritable and acquired vitamin K deficiency (antibiotics, rodenticide, and cholestyramine) [[5], [6], [7], [8]] and liver dysfunction, 2) hereditary bleeding disorders, 3) antithrombotic therapy usage, 4) disseminated intravascular coagulation, and 5) sepsis.

This study also included 50 control participants with bleeding, an age- and sex-matched group of children (*n* = 50; 23 males and 27 females; mean age, 6.14 years), who fulfilled the following inclusion criteria: 1) normal coagulation function, 2) normal liver and kidney function, 3) normal coagulation factor activity (including FVIII:C, FIX:C, FXI:C, FXII:C, FII:C, FV:C, FVII:C, and FX:C), and 4) without hereditary bleeding disorders.

### 2.2 Clinical information

All potential subjects underwent clinical evaluation. The clinical information of bleeding or thrombosis was recorded using standardized forms, including the presence or absence of bleeding symptoms, type of bleeding, quantity of bleeding, bleeding frequency, and the level of medical attention and treatment required for each. Follow-up was conducted by inpatient/outpatient medical history, by telephone questionnaire, and by interview from their parents. The time of diagnosis was determined when patients exhibited prolonged PT and aPTT, decreased FII:C, and LA positivity at the first visit to the outpatient. And, the interviews were conducted from the time of diagnosis till the time when LA turned negative with PT and FII:C levels completely returned to the normal range. The follow-up period was from the first diagnosis date to the last follow-up date in monthly units, and the last follow-up was on May 1, 2024.

Clinical bleeding symptoms were quantified (bleeding score) using the consensus International Society on Thrombosis and Haemostasis bleeding assessment tool (ISTH-BAT) [9,10]. The ISTH-BAT bleeding scores were compared with bleeding scores determined from a group of control subjects (*n* = 50) using the Mann–Whitney U-test. All participants gave written informed consent prior to any study procedures. All procedures were conducted in accordance with the Declaration of Helsinki and were approved by the Ethics Committee of Children’s Hospital, Zhejiang University School of Medicine with the ethics board number 2023-IRB-0114-P-01.

### 2.3 Coagulation tests

PT, aPTT, and thrombin time (TT) were detected by the 1-stage clotting method, and fibrinogen (Fg) was tested by the Clauss method. All coagulation factors’ activity levels (FVIII:C, FIX:C, FXI:C, FXII:C, FII:C, FV:C, FVII:C, and FX:C) were assessed by standard 1-stage clotting assays. Nijmegen assay was used to detect the specific coagulation factor (VIII and IX) inhibitors [11]. In the study, the normal reference values for all coagulation tests in children: PT (9.0-14.0 seconds), aPTT (23.0-38.0 seconds), TT (15.0-22.0 seconds), Fg (1.8-4.0 g/L), FVIII:C (50%-150%), FIX:C (50%-150%), FXI:C (50%-150%), FXII:C (50%-150%), FII:C (50%-150%), FV:C (50%-150%), FVII:C (50%-150%), and FX:C (50%-150%). All these assays were performed by CS-5100 automated coagulation analyzer (Sysmex Corporation). All operations were following the manufacturer’s protocols.

### 2.4 aPTT mixing study

aPTT mixing test is often used to estimate whether the prolongation is due to factor inhibitors (anticoagulation factors antibodies, or LA) or factor deficiency [12,13]. According to the standard procedures [13], aPTT mixing test utilizes 1:1 mixtures of patient plasma (PP) + normal pooled plasma (NPP), and then aPTT was tested multiple times (including immediate mixing and incubated at 37 ℃ for 2 hours mixing) to assess the degree of aPTT “correction” in mixed plasma.

We used the Rosner index (RI) as an index of circulating anticoagulant, with the cutoff value ranging from 10% to 15%. In general, RI of <10% indicates that the prolonged aPTT could be corrected with a 1:1 mixture of PP and NPP, suggesting the presence of factor deficiency; whereas RI of >15% indicates that the prolonged aPTT could not be corrected with a 1:1 mixture of PP and NPP, suggesting the presence of factor inhibitors (anticoagulation factors antibodies or LA). Moreover, both RI (immediate) of >15% and RI (2 hours) of >15% suggest the presence of LA but the absence of anti-FVIII Ab (time and temperature dependent antibodies) [[14], [15], [16]].

We collected NPP that would comprise a minimum of 20 normal individuals (equal ratio male/female) with normal coagulation function; platelet counts in platelet-poor plasma should be <10 × 10^9^/L.

### 2.5 Multidilution assay

Multidilution assay (MDA) is used as a test for screening whether decreased coagulation factor activity is due to the presence of inhibitors (anticoagulation factors antibodies or LA) or factor deficiency [[17], [18], [19]]. To perform MDA, standard curves of up to 6 points are processed, and samples (coagulation factor activity) are tested selecting the appropriate MDA dilution series (eg, doubling dilutions 1/2-1/128) using CS-5100 automated coagulation analyzer. Meanwhile, NPP was used as normal control. First, NPP was diluted by 1:1, 1:2, 1:4, 1:8, 1:16, and 1:32, and then, the coagulation factor activity (FVIII:C, FIX:C, FXI:C, FXII:C, FII:C, FV:C, FVII:C, FX:C) of NPP with different dilution ratio was evaluated by MDA.

In general, in the presence of factor deficiency, MDA line (factor activity and coagulation time response curve) is parallel to the standard curve, that is parallelism, which means that all coagulation factors’ activities return to the same level by multiplying the dilution ratio, whereas in the presence of factor inhibitors (anticoagulation factors antibodies or LA), MDA line is not parallel to the standard curve, that is nonparallelism, which means that decreased coagulation factor activity increases with the increase of dilution ratio and return to normal levels [[17], [18], [19]]. Taking the series of dilution as the X-axis, coagulation factor activity as the Y-axis, draw the curve to evaluate the parallelism.

### 2.6 Detection of LA

The diluted Russell’s viper venom time (DRVVT) [20] method was used to detect LA by using CS-5100 automated coagulation analyzer. DRVVT method should be performed following 3 steps: screening, mixing, and confirmatory studies. Samples with LA-screen and LA-confirm (S/C) ratios of ≥1.2 were defined as LA-positive [20].

### 2.7 Detection of autoantibodies

Antinuclear antibodies and anti–extracted nuclear antigens autoantibodies were measured with the dot enzyme-linked immunosorbent assay (EUROIMMUN Diagnostics). And, aPS/PT antibodies were measured with enzyme-linked immunosorbent assay (Mlbio).

### 2.8 Statistical analysis

Normally distributed variables were presented as mean ± SD, while nonnormally distributed variables were presented as medians and IQR (25th-75th percentiles). Normally distributed variables were analyzed by independent sample t test, while nonnormally distributed variables were analyzed by the nonparametric Mann–Whitney U-test. Categorical variables were compared with use of chi-squared analysis. The correlation between PT and the bleeding score with FII:C was examined using Spearman correlation coefficients. Data were analyzed using SPSS 20.0 (SPSS), and a 2-sided *P* value of <.05 was considered statistically significant.

## 3 Results

### 3.1 Demographic and clinical manifestations

The study included 70 patients with LAHPS, including 32 boys and 38 girls, with a mean age of 5.58 years (range: 0.67-13 years). Thirty-six (51.4%) subjects with LAHPS were first identified during the investigation of bleeding, 25 (35.7%) during the physical examination, and 9 (12.9%) at the preoperative clotting screening. These surgeries included adenotonsillectomy (*n* = 4), circumcision (*n* = 2), indirect inguinal hernia hernioplasty (*n* = 2), and distal radius fracture surgery (*n* = 1).

Among the 70 LAHPS cases, 51 (72.9%) were secondary to infection, 15 (21.4%) were secondary to autoimmune disease (AID), and 4 (5.7%) were due to unknown causes (Table 1). AID-associated LAHPS comprised 8 (11.4%) systemic lupus erythematosus (SLE), 3 (4.3%) mixed connective tissue diseases, 2 (2.9%) juvenile idiopathic arthritis, 1 (1.4%) anaphylactoid purpura, and 1 (1.4%) immune thrombocytopenic purpura cases. On the other hand, infection-associated LAHPS comprised 12 (17.1%) influenza A, 11 (15.7%) adenovirus (ADV), 11 (15.7%) SARS-CoV-2, 7 (10%) mycoplasma pneumoniae (MP), 2 (2.9%) MP and influenza A coinfection, 1 (1.4%) MP and respiratory syncytial virus coinfection, 1 (1.4%) MP and ADV coinfection, 1 (1.4%) cytomegalovirus, 1 (1.4%) influenza B, and 4 (5.7%) virus infection without documented pathogen cases (Supplementary Table S1).

**Table 1:** Demographic and clinical characteristics of 50 control subjects and 70 patients with lupus anticoagulant-hypoprothrombinemia syndrome.

Parameters	Control subjects (*n* = 50)	Total populations with LAHPS (*n* = 70)	Those LAHPS with bleeding/thrombosis (*n* = 57)	Those LAHPS without bleeding/thrombosis (*n* = 13)	*P* value
Race/ethnicity, *n* (%)					
Asian/Chinese	50 (100)	70 (100)	57 (100)	13 (100)	
Sex, *n* (%)					.55
Male	23 (46)	32 (45.7)	25 (43.9)	7 (53.8)	
Female	27 (54)	38 (54.3)	32 (56.1)	6 (46.2)	
Age (y), mean ± SD	6.14 ± 3.43	5.58 ± 2.82	5.35 ± 2.82	6.59 ± 2.88	.15
Associated disease, *n* (%)					<.99
Autoimmune disease	N/A	15 (21.4)	12 (21.1)	3 (23.1)	
Infection	N/A	51 (72.9)	41 (71.9)	10 (76.9)	
Unknown cause	N/A	4 (5.7)	4 (7)	0 (0)	
Bleeding episodes, *n* (%)	50 (100)	56 (80)	56 (98.2)	0	<.0001
Thrombosis, *n* (%)	0 (0)	1 (1.4)	1 (1.8)	0	<.99
ISTH-BAT bleeding score, median (IQR)	1 (1-2)	4 (2-4)	4 (3-4)	0	<.0001
Laboratory characteristics					
PT (s), median (IQR) or mean ± SD	11.5 ± 0.77	16.25 (15.28-17.60)	16.4 (15.40-17.70)	15.8 (14.8-16.55)	.13
aPTT (s), mean ± SD	28.6 ± 3.07	69.66 ± 21.16	70.16 ± 20.86	67.45 ± 17.34	.67
TT (s), mean ± SD	19.3 ± 1.0	19.1 ± 1.34	19.15 ± 1.40	18.91 ± 1.03	.58
Fg (g/L), mean ± SD	2.5 ± 0.62	2.6 ± 0.66	2.53 ± 0.66	2.91 ± 0.57	.06
FVIII^a^ (%), median (IQR) or mean ± SD	107.8 ± 16.5	12.55 (8.15-21.25)	12.6 (7.8-21.2)	10.5 (9.7-22.35)	.92
FIX^a^ (%), median (IQR) or mean ± SD	74.6 ± 20.3	7.15 (3.68-13.60)	7.1 (3.65-13.6)	8.9 (3.55-13.2)	.80
FXI^a^ (%), median (IQR) or mean ± SD	85.9 ± 15.1	8.05 (3.78-14.75)	8.2 (3.45-14.8)	6.4 (4.45-13.95)	.86
FXII^a^ (%), median (IQR) or mean ± SD	51.7 ± 9.3	5.65 (3.40-10.18)	5.7 (3.15-10.85)	5.5 (3.90-8.1)	.90
FII (%), mean ± SD	85.7 ± 18.3	23.38 ± 7.47	22.34 ± 7.53	27.96 ± 5.32	.01
FV (%), median (IQR) or mean ± SD	93.5 ± 17.1	90.6 (80.23-107.65)	95.3 (80-110.35)	89.2 (81.55-99.65)	.44
FVII (%), mean ± SD	80.1 ± 16.2	81.51 ± 16.02	80.84 ± 16.11	84.44 ± 15.89	.47
FX (%), mean ± SD	84.4 ± 16.7	78.85 ± 14.98	84.27 ± 17.65	84.27 ± 17.65	.15
S/C value (LA), median (IQR)	N/A	1.8 (1.61-2.18)	1.79 (1.63-2.17)	1.88 (1.54-2.38)	.92
LA turn negative (d), mean ± SD	N/A	31 ± 14	32 ± 14	28 ± 14	.35
aPTT mix testing					
RI (%) (immediate), mean ± SD	N/A	62.98 ± 22.06	61.67 ± 21.46	68.74 ± 24.67	.32
RI (%) (incubated at 37 ℃ for 2 h), mean ± SD	N/A	64.53 ± 19.88	64.26 ± 20.33	65.71 ± 18.58	.82

*P* < .05 and *P* < .0001 (with symptoms vs without symptoms groups).

aPTT, activated partial thromboplastin time; F, factor; Fg, fibrinogen; ISTH-BAT, International Society on Thrombosis and Haemostasis bleeding assessment tool; LA, lupus anticoagulant; LAHPS, lupus anticoagulant-hypoprothrombinemia syndrome; N/A, not applicable; PT, prothrombin time; RI, Rosner index; S/C, LA-screen and LA-confirm ratios; TT, thrombin time.

a The results in the initial testing.

**Figure 1:**
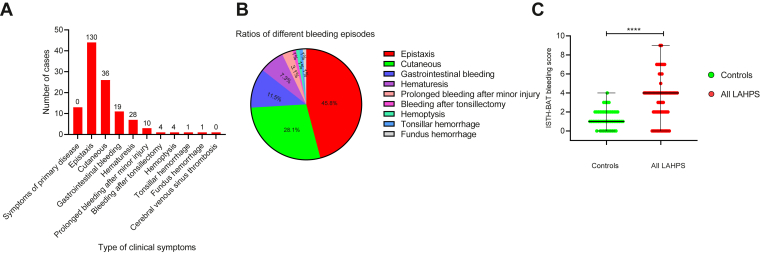
Clinical symptoms of the 70 patients with lupus anticoagulant-hypoprothrombinemia syndrome (LAHPS). (A) Number of cases of different clinical symptoms in patients with LAHPS. The number at the top of the bar chart refers to the total scores for different bleeding episodes; (B) ratios of different bleeding episodes in 56 patients; (C) bleeding scores in subjects with LAHPS were compared with scores from an age- and sex-matched control group. The top and the bottom lines represent the range of (International Society on Thrombosis and Haemostasis bleeding assessment tool [ISTH-BAT]) bleeding scores, and the middle line represents the median of (ISTH-BAT) bleeding score. ∗∗∗∗*P* < .0001.

The 70 patients were followed up for 3 to 8 months, with a median (IQR) follow-up time of 3 (3-3.25) months. No patients were lost or died during the follow-up period. 13 (18.6%) had only clinical symptoms of primary disease without bleeding, while 56 (80%) presented with bleeding, and 1 (1.4%) had suffered from cerebral venous sinus thrombosis (Table 1). A total of 96 bleeding episodes were recorded in 56 patients (bleeding score, ≥2), with a median ISTH-BAT bleeding score of 4 (range, 0-9). The most common bleeding symptom was spontaneous minor to moderate epistaxis (45.8%), followed by cutaneous (28.1%; subcutaneous hemorrhagic spot, ecchymosis, and purpura), gastrointestinal bleeding (11.5%; hematemesis and hematochezia), hematuresis (7.3%), prolonged bleeding after minor injury (3.1%; venipuncture, facial scratches, and lip laceration), bleeding after tonsillectomy (1.0%), hemoptysis (1.0%), tonsillar hemorrhage (1.0%), and fundus hemorrhage (1.0%) (Figure 1A, B). Furthermore, there were 44 (62.9%) patients with ISTH-BAT bleeding score of ≥3.

All 50 controls exhibited normal PT (11.5 ± 0.77 seconds; range, 10.1-13.5 seconds), normal aPTT (28.6 ± 3.07 seconds; range, 25.4-36.5 seconds), normal TT (19.3 ± 1.0 seconds; range, 17.4-23.6 seconds), normal Fg (2.5 ± 0.62 g/L; range, 1.62-3.97 g/L), and normal 8 coagulation factors’ activities (FVIII:C, 107.8% ± 16.5%; FIX:C, 74.6% ± 20.3%; FXI:C, 85.9% ± 15.1%; FXII:C, 51.7% ± 9.3%; FII:C, 85.7% ± 18.3%; FV:C, 93.5% ± 17.1%; FVII:C, 80.1% ± 16.2%; FX:C, 84.4% ± 16.7%; Table 1). A total of 65 bleeding episodes occurred in 50 control subjects, and each subject experienced bleeding at least at 1 anatomical site, including epistaxis (*n* = 39), ecchymosis (*n* = 13), menorrhagia (*n* = 10), and gastrointestinal hemorrhage (*n* = 3). The overall bleeding scores in subjects with LAHPS (median, 4; IQR, 2-4; range, 0-9) were higher than those in matched control subjects with normal coagulation functions (median, 1; IQR, 1-2; range, 0-4; *P* < .0001; Figure 1C).

### 3.2 Laboratory characteristics of patients with LAHPS

Thrombocytopenia was observed in 4 patients with AID (Supplementary Table S1), and 66 subjects displayed normal platelet counts (mean, 249 × 10^9^/L) and normal platelet function. Several autoantibodies were identified in 62 patients, and no autoantibodies test was performed in 8 patients. These autoantibodies include aPT antibodies (*n* = 55), anti–double stranded DNA antibodies (*n* = 10), anti-U1-ribonucleoprotein antibodies (*n* = 6), anticardiolipin antibodies (*n* = 6), antihistone antibodies (*n* = 4), anti-Smith antibodies (*n* = 4), anti-Sjogren syndrome A antibodies (*n* = 4), antinuclear antibodies (*n* = 3), rheumatoid factors (*n* = 2), antiplatelet antibodies (*n* = 4), anti-FVIII Ab (*n* = 2), anti-β2 glycoprotein antibodies (*n* = 2), anti-Sjogren syndrome B antibodies (*n* = 1), antinucleosome antibodies (*n* = 1), anti–scleroderma-70 antibodies (*n* = 1), anti-p-neutrophil cytoplasmic antibodies (*n* = 1), and anti–cyclic citrullinated peptide antibodies (*n* = 1), respectively.

**Figure 2:**
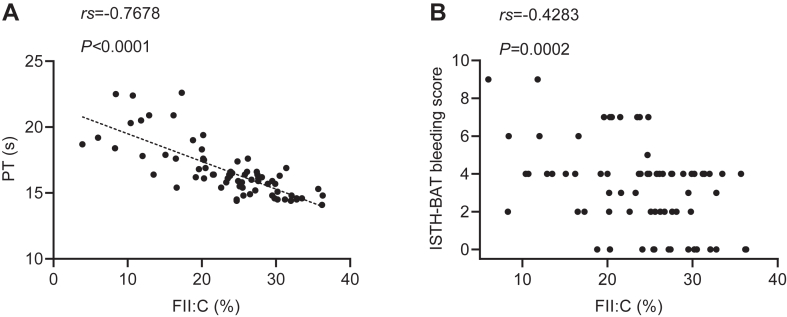
Correlation analysis of factor II activity (FII:C) with prothrombin time (PT) and International Society on Thrombosis and Haemostasis bleeding assessment tool (ISTH-BAT) bleeding score. The relationship of coagulation FII:C with (A) PT and (B) ISTH-BAT bleeding score.

All subjects exhibited prolonged PT (median, 16.25 seconds; range, 14.3-22.6 seconds), significantly prolonged aPTT (mean, 69.68 seconds; range, 40.4-118 seconds), and decreased FII:C (mean, 23.38%, range, 3.9%-36.3%). All patients exhibited normal TT (mean, 19.1 seconds; range, 14.8-22.6 seconds), normal Fg (mean, 2.6 g/L; range, 1.65-4.9 g/L), normal FV:C (median, 90.6%; range, 56.8%-167.6%), normal FVII:C (mean, 81.51%; range, 51.1%-115.4%), and normal FX:C (mean, 78.85%; range, 45.7%-111.8%). Furthermore, all subjects showed decreased FVIII:C (median, 12.55%; range, 1.7%-33.8%), decreased FIX:C (median,7.15%; range, 0.6%-27.2%), decreased FXI:C (median, 8.05%; range, 0.5%-24.0%), and decreased FXII:C (median, 5.65%; range, 0.7%-26.8%) in the initial testing. The levels of FII:C in patients with bleeding were lower than those without bleeding (22.34% ± 7.53% vs 27.96% ± 5.32%; *P* = .01; Table 1). No significant differences were observed for other variables between patients with and without symptoms (*P* > .05; Table 1). Correlation analysis revealed that there was a significant negative correlation between FII:C and PT (*rs* = −0.7678; *P* < .0001; Figure 2A), while the severity of bleeding was weakly correlated with FII:C (*rs* = −0.4283; *P* < .001; Figure 2B).

**Figure 3:**
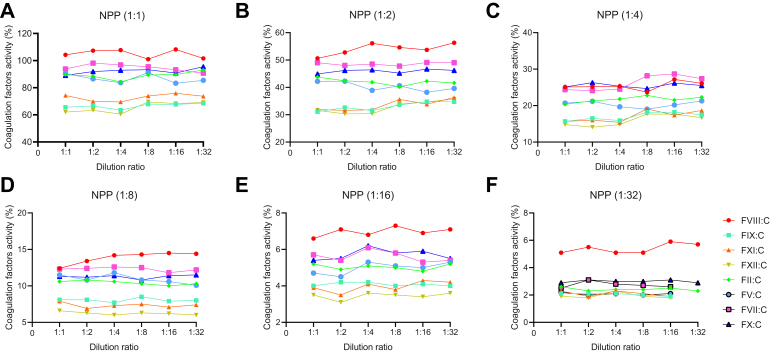
The parallelism of coagulation factors activity in normal pooled plasma (NPP) using multidilution assay. Eight coagulation factors activities in NPP returns to the same level by multiplying the dilution ratio, regardless of the dilution ratio of the original plasma sample: (A) 1:1; (B) 1:2; (C) 1:4; (D) 1:8; (E) 1:16; (F) 1:32.

**Figure 4:**
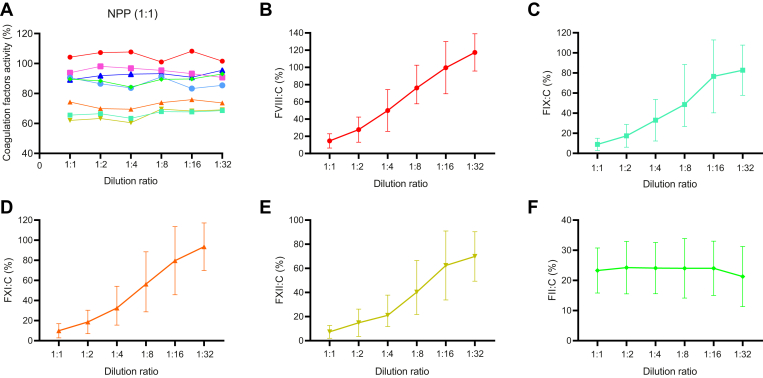
The nonparallelism of coagulation factors activity in 70 patients with lupus anticoagulant-hypoprothrombinemia syndrome using multidilution assay. (A) Eight coagulation factors activity in normal pooled plasma (NPP) returns to the same level by multiplying the dilution ratio; (B–E) decreased endogenous coagulation factors activity (FVIII:C, FVIX:C, FXI:C, and FXII:C) in 70 lupus anticoagulant-hypoprothrombinemia syndrome patients plasma increased with the increase of dilution ratio and returned to normal levels; (F) FII:C did not increase with the increase of dilution and still remained at a low level. (B–F) The top and the bottom lines represent SD, and the different shapes of the boxes in the middle refer to mean level of different coagulation factors’ activities.

In 65 subjects (5 patients did not undergo aPTT mixing test), 96.9% (63/65) patients showed that both immediate and incubated for 2 hours at 37 °C of aPTT mixing test were uncorrected (RI, >15%), with the mean RI of 62.98% (range, 22.9%-96.6%) and 64.53% (range, 24.1%-99.0%), respectively. As shown in Figure 3A–F, MDA showed that all coagulation factors’ activities return to the same level by multiplying the dilution ratio in NPP, that is parallelism. Whereas, in 70 PPs, the initial testing for FVIII:C, FIX:C, FXI:C, and FXII:C showed evidence of inhibition but corrected with MDA serial dilutions, that is nonparallelism. The nonparallelism showed that endogenous coagulation factor activity increased with the increase of dilution ratio and returned to normal levels (Figure 4B–E). And, FII:C did not increase with the increase of dilution and still remained at a low level (Figure 4F).

The DRVVT method showed that positive LA was identified in all subjects, with the median S/C value of 1.8 (range, 1.23-3.23; S/C value in 3 patients was not recorded). Both LA and anti-FVIII Ab (2.3 BU and 1.03 BU) were detected in 2 patients (1 SLE and 1 connective tissue diseases), who exhibited uncorrected aPTT (immediately and incubated at 37 ℃ for 2 hours). Notably, LA in all patients turned negative within 12 weeks (mean, 31 days; range, 8-70 days).

### 3.3 Comparison of clinical characteristics between AID- and infection-associated LAHPS

**Figure 5:**
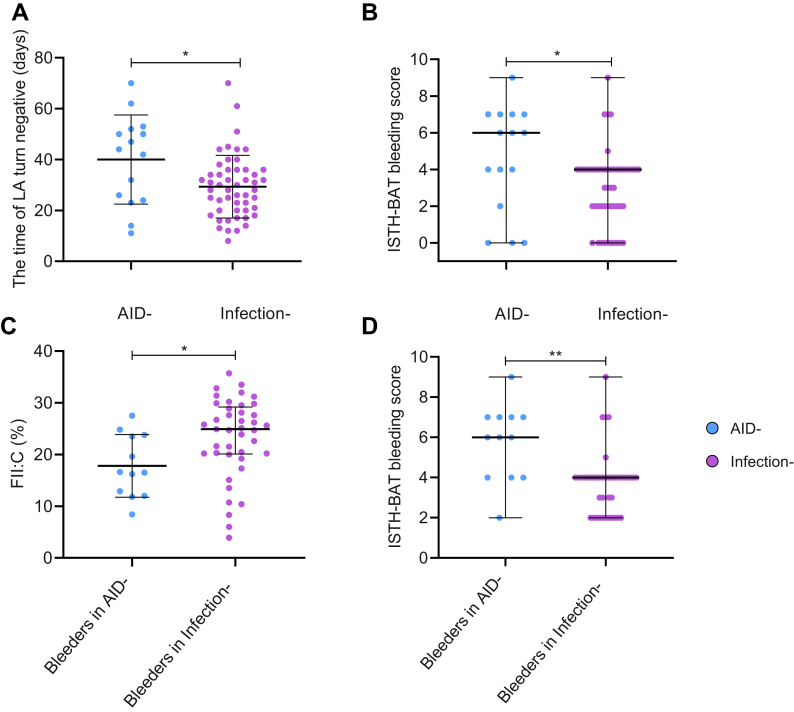
Comparison of clinical features in patients with autoimmune disease (AID)–associated and infection-associated lupus anticoagulant-hypoprothrombinemia syndrome. The top and the bottom lines represent SD, and the middle line represents mean values in (A) and (C); the top and the bottom lines represent the range of (International Society on Thrombosis and Haemostasis bleeding assessment tool [ISTH-BAT]) bleeding scores, and the middle line represents the median of (ISTH-BAT) bleeding score in (B) and (D). ∗*P* < .05; ∗∗*P* < .01 (AID-associated vs infection-associated lupus anticoagulant-hypoprothrombinemia syndrome).

The mean age of patients with infection-associated LAHPS (4.81 ± 2.23 years) was lower than that of patients with AID-associated LAHPS (8.06 ± 2.96 years; *P* < .001). The mean time of LA turn negative in infection group (29 ± 12 days) was shorter than that in the AID group (40 ± 17 days; *P* = .04; Figure 5A). The mean level of FII:C in AID group (20.49% ± 7.92%) was slightly lower than that in infection group (23.99% ± 7.39%; *P* = .12), whereas the median ISTH-BAT bleeding scores in AID group (median, 6; IQR, 4-7; range, 0-9) were higher than in infection group (median, 4; IQR, 2-4; range, 0-9; *P* = .03; Figure 5B). Additionally, the mean level of FII:C of bleeders was lower in AID group (17.8% ± 6.05%) than that in infection group (23.26% ± 7.68%; *P* = .01; Figure 5C), whereas the median ISTH-BAT bleeding scores of bleeders in AID group (median, 6; IQR, 4-7; range, 2-9) were higher than that in infection group (median, 4; IQR, 2-4; range, 2-9; *P* = .001; Figure 5D). No significant differences were observed for other variables between the 2 groups. The results are shown in Table 2.

**Table 2:** Demographic and clinical characteristics of patients with autoimmune disease–associated and infection-associated lupus anticoagulant-hypoprothrombinemia syndrome.

Parameters	AID (*n* = 15)	Infection (*n* = 51)	*P* value
Age (y), mean ± SD	8.06 ± 2.96	4.81 ± 2.23	<.001
Sex, *n* (%)			.02
Male	3 (20)	27 (52.9)	
Female	12 (80)	24 (47.1)	
Bleeding episodes, n (%)	12 (80)	41 (80.4)	.97
ISTH-BAT bleeding score, median (IQR)	6 (4, 7)	4 (2, 4)	.03
ISTH-BAT bleeding score of bleeders, median (IQR)	6 (4-7)	4 (2-4)	.001
Laboratory characteristics			
PT (s), median (IQR)	16.8 (14.5-20.5)	15.9 (14.8-21.4)	.28
aPTT (s), mean ± SD	68.43 ± 16.87	70.67 ± 21.42	.71
TT (s), mean ± SD	19.20 ± 1.39	19.10 ± 1.35	.81
Fg (g/L), mean ± SD	2.58 ± 0.54	2.63 ± 0.71	.78
FVIII:C^a^ (%), median (IQR)	14.1 (8.0-18.3)	12.5 (3.9-7.2)	.74
FIX:C^a^ (%), median (IQR)	6.2 (3.1-13.2)	7.2 (3.9-13.6)	.50
FXI:C^a^ (%), median (IQR)	8.1 (6.4-13.1)	9.6 (4.1-15.2)	.05
FXII:C^a^ (%), median (IQR)	5.1 (3.4-7.1)	6.4 (3.2-11.3)	.49
FII:C (%), mean ± SD	20.49 ± 7.92	23.99 ± 7.39	.12
FV:C (%), median (IQR)	89.3 (81.1-106)	94.9 (79.8-108.7)	.75
FVII:C (%), mean ± SD	77.34 ± 16.31	82.78 ± 16.05	.25
FX:C (%), mean ± SD	72.76 ± 14.01	80.44 ± 15.27	.08
FII:C (%) of bleeders, mean ± SD	17.8 ± 6.05	23.26 ± 7.68	.01
S/C value (LA), median (IQR)	1.93 (1.61-2.47)	1.77 (1.6-2.08)	.14
LA turn negative (d), mean ± SD	40 ± 17	29 ± 12	.04

*P* < .05, *P* < .001, and *P* < .0001 (AID vs infection groups).

AID, autoimmune disease; aPTT, activated partial thromboplastin time; Fg, fibrinogen; FII:C, factor II activity; FIX:C, factor IX activity; FV:C, factor V activity; FVII:C, factor VII activity; FVIII:C, factor VIII activity; FX:C, factor X activity; FXI:C, factor XI activity; FXII:C, factor XII activity; ISTH-BAT, International Society on Thrombosis and Haemostasis bleeding assessment tool; LA, lupus anticoagulant; PT, prothrombin time; S/C, LA-screen and LA-confirm ratios; TT, thrombin time.

a The results in the initial testing.

### 3.4 Treatments and outcomes

In our study, 24 (34.3%) subjects with or without bleeding only received treatment for primary disease, but did not receive any coagulation factor replacement therapy and corticosteroids therapy. And, 1 patient with cerebral venous sinus thrombosis received anticoagulated therapy. In addition, 4 (5.7%) patients without bleeding were treated with oral or intravenous corticosteroids (methylprednisolone) alone, and 16 (22.9%) patients were treated with a combination of corticosteroids with an immunosuppressor (cyclophosphamide, azathioprine, methotrexate, tocilizumab, hydroxychloroquine). Furthermore, 3 (4.3%) patients were treated with a combination of methylprednisolone with intravenous immunoglobulin with good outcome (1 SLE, 1 immune thrombocytopenic purpura, and 1 ADV enteritis). Additionally, 1 patient with juvenile idiopathic arthritis–associated LAHPS received methotrexate combined with adalimumab after treated with recombinant human tumor necrosis factor-α receptor Ⅱ:IgG-Fc fusion protein. Notably, a total of 144 replacement treatment records were collected from 41 (58.6%) patients for prevention and control of bleeding, or for surgical prophylaxis (*n* = 11, 4 adenotonsillectomy, 2 circumcision, 2 indirect inguinal hernia hernioplasty, 1 distal radius fracture surgery, 1 bone marrow puncture, 1 appendectomy). All replacement treatments include 63 vitamin K (intramuscular injection, 3-10 mg/day), 28 fresh frozen plasma transfusions with a median volume of 190 mL (range, 120-400), 22 prothrombin complex concentrates venous transfusion (200-400 IU), 20 etamsylate (0.25-0.5 g; taken orally 10 mg/kg or intravenous drip 30 mg/kg), 3 hemostatic pump packings, 2 sutures, 2 transfusions of suspended red blood cells, and 1 hemostasis by burning.

Overall, FII:C levels were lower in patients who needed treatment (*n* = 56) to prevent or control bleeding (22.15% ± 7.46%) than in those who did not (*n* = 14; 28.31% ± 5.27%; *P* = .01), with the optimal FII:C cutoff level of 25.25%. After followed up, 65 (92.9%) patients had a complete remission, and 5 (7.1%) patients with AID presented with partial remission. And, PT and FII:C were completely normalized of all patients without recurrence of bleeding and without thrombosis.

## 4 Discussion

Thrombosis is a characteristic manifestation of antiphospholipid syndrome [21,22]. In contrast, LAHPS is associated with a bleeding tendency [2,4,23,24]. The proposed mechanism of bleeding in LAHPS is that aPT or aPS/PT antibodies bind prothrombin (FII) without neutralizing its activity, resulting in rapid clearance of these prothrombin-antibody immune complexes by the reticuloendothelial system [25]. Several studies have reported that LAHPS is usually associated with AIDs [[26], [27], [28]], or an infection [4], or tumor [24,29], and it is more frequent in the pediatric population and female gender.

We have reported 70 pediatric patients diagnosed with LAHPS at a single center, with boys–to-girls ratio being 1:1.20. In our study, most of (72.9%) LAHPS patients were related to infection, followed by AIDs (21.4%). All 70 subjects exhibited prolonged PT, significantly prolonged aPTT, LA positivity, and decreased FII:C, which aligns with the previously proposed laboratory diagnosis for LAHPS [2,26,28,30].

In our study, the prolonged aPTT could not be corrected with a 1:1 mixture of PP and NPP (immediately and incubated at 37 ℃ for 2 hours), suggesting the presence of factor inhibitor (ie, LA). Notably, in all LAHPS subjects, MDA showed that the decreased levels of endogenous coagulation factors’ activities increased with the increase of dilution ratio and returned to normal levels, suggesting the presence of LA. This nonparallelism is attributed to the ability of LA to inhibit phospholipid-dependent clotting times in a nonfactor specific manner that was weakened with the increase of dilution, leading to the unbalanced optimal ratio of antigen-antibody response [31]. Whereas, MDA showed that FII:C in all subjects was still significantly decreased, regardless of dilution ratio. As previously reported [17,32,33], nonparallelism was observed in aPTT-based factors (FVIII, FIX, FXI, and FXII) but not in PT/common pathway-based factors (FII, FV, FVII, and FX). This is possibly due to the high concentration of phospholipid in PT reagents [34].

Clinical manifestations of LAHPS are highly heterogeneous, ranging from bleeding [2,24,30] to thrombotic events, or a combination of both [35], although many patients remain asymptomatic [32]. The main clinical association of LAHPS was bleeding, which was identified in 80% subjects in our study. Moreover, 25.7% of patients had bleeding at more than 2 anatomic sites, and 62.9% of subjects had a bleeding score ≥3. Overall, subjects with LAHPS showed that bleeding scores were higher than those described for patients with other heritable bleeding disorders, including congenital Fg disorders [36,37], FXI deficiency [38], von Willebrand disease [39], and platelet function disorders [39]. Meanwhile, we found that the severity of bleeding symptoms was weakly correlated with the decreased level of FII:C (*rs* = −0.4283; *P* < .001). The overall bleeding scores in AID group were higher than those in infection group (*P* < .05). These data indicated that besides hypoprothrombinemia, local vascular inflammation, acquired FVIII inhibitors and thrombocytopenia are also the causes of LAHPS bleeding [40,41], which may explain the above result. And, thrombosis occurred rarely in the study, which was identified in 1.4% included subjects.

Currently, there is no standard or consensus on the treatment of LAHPS. In our study, treatment for LAHPS patients included treatment for primary disease, clearing LA and autoantibodies, and prevention and control of bleeding. Oral or intravenous corticosteroids were considered as a first-line treatment for clearance of LA and autoantibodies, and/or in combination with immunosuppressants or intravenous immunoglobulin. Replacement therapy (vitamin K, fresh frozen plasma, prothrombin complex concentrate, etamsylate, and tranexamic acid) was efficacious for on-demand treatment of bleeding and surgical prophylaxis. In our study, patients who needed therapy have lower FII:C levels (*P* < .01) compared with those who did not. We recommended that coagulation factors replacement therapy should be properly considered for LAHPS patients with FII:C of ≤25.25% and for patients undergoing surgery especially at highly fibrinolytic activity sites. It is worth noting that treatment aimed at correcting coagulation FII deficiency may promote thromboembolism, caution must be used in selecting which patient to treat and duration of therapies. The limitation of the study was that the patients included in this study came as outpatients and some patients were unable to provide details of their local hospital treatment during the follow-up. And, because the study included 70 patients from a single center in the study, further clinical data from multicenter studies are needed to optimize the treatment guidelines for patients with LAHPS.

## 5 Conclusion

Pediatric patients with LAHPS most commonly develop LAHPS after AIDs and infection, especially preschool patients (younger than 5 years) with infection. Most patients presented with mild to moderate bleeding. In the absence of lupus anticoagulant testing, MDA method combined with aPTT mixing test and FII:C can improve the feasibility and accuracy of diagnosis of LAHPS, and provide a reliable laboratory basis for early clinical diagnosis and treatment of LAHPS.
