# Supplementary material for: Clinical features and treatment of 70 children with lupus anticoagulant-hypoprothrombinemia syndrome: a retrospective study from a single center in China
Source: Res Pract Thromb Haemost. 2024 Sep 26;8(7):102577. doi: 10.1016/j.rpth.2024.102577 (PMC11513820; doi:10.1016/j.rpth.2024.102577)
Supplement: Supplementary Table S1 [file mmc1.doc]

Table SI Demographic data, clinical symptoms, and laboratory characteristics of 70 patients with lupus anticoagulant-hypoprothrombinemia syndrome

| Case | Sex | Age  (y) | PT  (s) | APTT  (s) | Fg  (g/L) | TT  (s) | FVIII:  C (%) | FIX:  C (%) | FXI:  C (%) | FXII:  C (%) | FII:  C (%) | FV:  C (%) | FVII:  C (%) | FX:  C (%) | APTT  mixing test  (corrected) | MDA ^a^ | PLT  ×10^9/L | S/C  value  (LA) | Associated primary  disease | Clinical symptoms with bleeding | Medical attention  or treatment ^c^ | ISTH  Bleeding scores |  |
| --- | --- | --- | --- | --- | --- | --- | --- | --- | --- | --- | --- | --- | --- | --- | --- | --- | --- | --- | --- | --- | --- | --- | --- |
| 1 | M | 5.25 | 14.8 | 49.4 | 1.95 | 18.7 | 25.6 | 13.6 | 8.6 | 18 | 25.6 | 105.4 | 108 | 1.95 | No | Non- | 430 | 1.23 | SARS-CoV-2 | Hemorrhagic spots | Consultation | 2 |  |
| 2 | F | 10 | 20.5 | 57 | 3.09 | 18.7 | 26 | 15.2 | 16.8 | 5.6 | 11.8 | 81.1 | 65.3 | 3.09 | No | Non- | 233 | 2.45 | SLE | Hemorrhagic spots,  Bruises, Epistaxis,  Hematuria | Extensive,  FFP,  FFP | 9 |  |
| 3 | M | 3.42 | 15.9 | 76.4 | 3.09 | 18.2 | 24.7 | 9.8 | 12 | 5.5 | 29.5 | 82.7 | 115.4 | 3.09 | No | Non- | 179 | 1.57 | ADV | A | Untreated | 0 |  |
| 4 | F | 11 | 20.9 | 92.4 | 3.98 | 17.3 | 12 | 6.2 | 4.9 | 3.4 | 16.2 | 71.9 | 51.4 | 3.98 | No | Non- | 182 | 3.23 | SLE | Epistaxis | PCC, FFP, Etamsylate | 4 |  |
| 5 | F | 5.33 | 17.4 | 58.2 | 2.59 | 20.4 | 6.5 | 2.5 | 4.8 | 4.6 | 24.8 | 84.1 | 51.1 | 2.59 | No | Non- | 153 | 1.82 | RA | Purpura,  Hematuria,  Bruises,  Hematochezia | VitK,  Etamsylate | 7 |  |
| 6 | F | 7 | 15.8 | 47.6 | 2.97 | 17.4 | 27 | 9.5 | 6.4 | 6.7 | 25.4 | 126.3 | 73.8 | 2.97 | No | Non- | 214 | 1.58 | ADV | A | Untreated | 0 |  |
| 7 | M | 8 | 15.4 | 56.2 | 2.94 | 17.4 | 14.1 | 10.3 | 6.9 | 9.1 | 16.6 | 115.1 | 73.7 | 2.94 | No | Non- | 38 | 2.18 | ITP | Bruises,  Epistaxis | VitK | 6 |  |
| 8 | F | 8 | 16.8 | 99.5 | 2.56 | 18.7 | 3.8 | 1.5 | 2.8 | 1.2 | 19.6 | 90.6 | 71.6 | 2.56 | No | Non- | 280 | 1.36 | MCTD | Bruises,  Epistaxis,  Hematochezia | VitK | 7 |  |
| 9 | F | 3.83 | 20.9 | 76.8 | 2.34 | 19.2 | 8 | 3.1 | 2 | 2 | 12.9 | 119.2 | 62.8 | 2.34 | No | Non- | 335 | 1.49 | JIA | Epistaxis,  Hemorrhagic spots | Consultation | 4 |  |
| 10 | M | 5.92 | 22.5 | 86.5 | 2.13 | 18.4 | 3.7 | 1.2 | 0.5 | 2.5 | 8.4 | 90.6 | 74.7 | 2.13 | No | Non- | 210 | 2.33 | MCTD | Epistaxis,  Bruises | VitK, Hemostatic pump | 6 |  |
| 11 | F | 3.83 | 18.7 | 53.4 | 2.43 | 18.9 | 5.3 | 2.2 | 2.9 | 4.2 | 3.9 | 150.5 | 101.1 | 2.43 | No | Non- | 164 | 2.04 | ADV | Epistaxis, | VitK, | 4 |  |
| 12 | F | 4.58 | 19.2 | 118 | 2.69 | 19.2 | 5 | 2 | 3 | 2 | 6 | 70.4 | 68 | 2.69 | No | Non- | 170 | 1.47 | Virus infection ^b^ | Epistaxis,  Bruises，  Hematochezia | FFP,  VitK,  PCC | 9 |  |
| 13 | M | 1.92 | 16.9 | 109.8 | 2.18 | 18.3 | 10.3 | 4.3 | 11.2 | 7.4 | 31.4 | 129.5 | 87 | 2.18 | No | Non- | 179 | 2.16 | SARS-CoV-2 | Epistaxis | FFP,  VitK,  Etamsylate | 4 |  |
| 14 | F | 4.92 | 20.3 | 117 | 1.8 | 21.2 | 5.5 | 1.5 | 3.1 | 1.8 | 10.4 | 116 | 71.8 | 1.8 | No | Non- | 358 | 1.86 | SARS-CoV-2 | Epistaxis | VitK | 4 |  |
| 15 | M | 6 | 16.0 | 108.8 | 2.06 | 20.5 | 8.4 | 7.1 | 8.2 | 7.7 | 26.7 | 107.3 | 77.1 | 2.06 | No | Non- | 239 | 2.42 | ADV | Epistaxis | Consultation | 2 |  |
| 16 | F | 5.58 | 16.4 | 77.3 | 1.95 | 20.7 | 5.9 | 3.9 | 5.3 | 2.9 | 25.8 | 103.5 | 109.3 | 1.95 | No | Non- | 165 | 1.67 | SARS-CoV-2 | Epistaxis | VitK | 4 |  |
| 17 | M | 3.08 | 14.5 | 50.7 | 2.69 | 17.7 | 14.6 | 10.4 | 14.9 | 6.4 | 32.8 | 80 | 74 | 2.69 | No | Non- | 327 | 1.68 | Virus infection ^b^ | Epistaxis,  Bruises | Consultation  ≥5 bruises (>1 cm) in exposed  areas | 3 |  |
| 18 | M | 4.17 | 14.4 | 108.2 | 2.27 | 20.7 | 7.5 | 5.6 | 7.2 | 3.5 | 24.7 | 79.6 | 87 | 2.27 | No | Non- | 375 | 2.08 | Flu A | Epistaxis | VitK | 4 |  |
| 19 | F | 2.67 | 14.9 | 71.5 | 3.44 | 16.5 | 12.4 | 9.3 | 14.7 | 5.7 | 26.5 | 167.6 | 90.3 | 3.44 | No | Non- | 195 | 1.81 | RSV＋MP | Epistaxis | VitK | 4 |  |
| 20 | F | 2 | 15.1 | 66.1 | 2.82 | 17.7 | 1.7 | 0.6 | 1 | 0.7 | 30.2 | 131.1 | 65.4 | 2.82 | No | Non- | 313 | 1.68 | Virus infection ^b^ | Epistaxis,  Hematuria | FFP,  Etamsylate | 4 |  |
| 21 | F | 2.25 | 16.4 | 48.7 | 2.04 | 16.8 | 10 | 3.6 | 4.2 | 3.8 | 23.7 | 103.3 | 58 | 2.04 | No | Non- | 175 | 1.58 | Unknown cause | Bruises | VitK | 4 |  |
| 22 | M | 4.17 | 16.4 | 49 | 1.78 | 18.4 | 25.9 | 27.2 | 14.2 | 13 | 21.6 | 101.6 | 70.8 | 1.78 | No | Non- | 135 | 1.59 | SARS-CoV-2 | Facial trauma | Suture | 3 |  |
| 23 | M | 5.92 | 15.3 | 70.8 | 3.02 | 19.4 | 17.3 | 12.7 | 14.9 | 8.7 | 35.7 | 136.7 | 62.3 | 3.02 | ND | Non- | 192 | 1.76 | MP＋ADV | Epistaxis | FFP | 4 |  |
| 24 | F | 3.08 | 18.3 | 58.5 | 3.74 | 17.4 | 14.8 | 12.9 | 15.7 | 9.4 | 20 | 121.2 | 109 | 3.74 | No | Non- | 323 | 1.80 | Flu A | Epistaxis | PCC,  VitK | 4 |  |
| 25 | M | 1.58 | 14.8 | 81.6 | 2.99 | 17.4 | 9.1 | 3.3 | 5 | 5.4 | 36.3 | 120.5 | 111.9 | 2.99 | No | Non- | 336 | 2.58 | MP | A | VitK | 0 |  |
| 26 | M | 6 | 16.1 | 90.4 | 4.37 | 17.4 | 12.5 | 4.6 | 6.1 | 1.6 | 20.3 | 108.7 | 53.5 | 4.37 | No | Non- | 225 | 2.72 | CMV | Epistaxis,  Hematemesis，  Hemorrhagic spots | FFP,  PCC,  VitK,  Etamsylate | 7 |  |
| 27 | M | 5.08 | 16.4 | 58.8 | 2.77 | 19.2 | 20.1 | 16.2 | 17.5 | 7.1 | 27.5 | 97.8 | 88.9 | 2.77 | No | Non- | 296 | 1.61 | JIA | Epistaxis | VitK | 4 |  |
| 28 | M | 4.67 | 16.1 | 51.7 | 2.56 | 20.1 | 18.9 | 6.1 | 7.8 | 12.3 | 27.6 | 101.8 | 70.4 | 2.56 | No | Non- | 203 | 1.78 | ADV | Hematuria | VitK,  FFP,  PCC | 4 |  |
| 29 | M | 0.75 | 19.4 | 41.7 | 4.9 | 14.8 | 10.8 | 12 | 11.8 | 10.1 | 20.2 | 73.2 | 56.5 | 4.9 | No | Non- | 189 | 1.58 | ADV | Hemorrhagic spots | VitK | 3 |  |
| 30 | F | 0.67 | 14.5 | 62.1 | 1.75 | 21.6 | 3.8 | 1.9 | 1.8 | 1.4 | 31.2 | 56.8 | 54.1 | 1.75 | ND | Non- | 316 | 2.41 | Flu A | Bleeding from  minor wounds | VitK | 4 |  |
| 31 | F | 7 | 15.4 | 66 | 2.11 | 18.8 | 21.4 | 15.4 | 18.2 | 16.4 | 22.6 | 96.9 | 77.6 | 2.11 | ND | Non- | 302 | 1.45 | SARS-CoV-2 | Epistaxis | Consultation | 2 |  |
| 32 | M | 5 | 16.2 | 57.2 | 2.36 | 19.1 | 33.8 | 13.6 | 18.2 | 11.8 | 19.2 | 118.5 | 93.3 | 2.36 | ND | Non- | 362 | 1.72 | ADV | Epistaxis | VitK | 4 |  |
| 33 | M | 5.92 | 16.4 | 58 | 2.22 | 18.1 | 29.2 | 11.8 | 14.6 | 11.9 | 13.5 | 94.9 | 83.3 | 2.22 | ND | Non- | 218 | 1.69 | Virus infection ^b^ | Epistaxis | FFP | 4 |  |
| 34 | F | 3.75 | 15.8 | 84 | 2.1 | 19.4 | 24.6 | 9 | 12.9 | 10.4 | 23.3 | 120.7 | 92.4 | 2.1 | No | Non- | 249 | 2.38 | Unknown cause | Hemorrhagic spots | VitK | 3 |  |
| 35 | F | 3.92 | 17.6 | 63.3 | 1.84 | 18.2 | 17.8 | 20.7 | 10.5 | 21.6 | 16.5 | 125.1 | 102.1 | 1.84 | No | Non- | 281 | 1.93 | MCTD | Epistaxis,  Bruises | Consultation | 2 |  |
| 36 | F | 3.5 | 18.4 | 62.9 | 2.65 | 21.6 | 9.3 | 5.9 | 9.7 | 4.2 | 8.3 | 63.1 | 51.5 | 2.65 | No | Non- | 328 | 2.01 | ADV | Epistaxis | Consultation | 2 |  |
| 37 | F | 13 | 17.8 | 75.2 | 2.35 | 22.6 | 15.5 | 5 | 4.2 | 7.1 | 12 | 106 | 103.3 | 2.35 | No | Non- | 37 | 2.56 | SLE | Hemorrhagic spots,  Hematuria,  Fundus hemorrhage | FFP,  PCC, | 6 |  |
| 38 | F | 8 | 14.3 | 71.7 | 2.43 | 18.3 | 10.3 | 3.8 | 8.3 | 6.4 | 36.2 | 80.4 | 91.6 | 2.43 | No | Non- | 310 | 2.47 | SLE | A | Untreated | 0 |  |
| 39 | F | 2.25 | 17.9 | 51.5 | 1.89 | 18.7 | 21.7 | 4.4 | 12.6 | 7 | 15.1 | 112 | 98 | 1.89 | No | Non- | 193 | 1.52 | SARS-CoV-2 | Epistaxis | VitK | 4 |  |
| 40 | F | 12.08 | 16.1 | 79.9 | 2.68 | 19.6 | 18.3 | 3.7 | 2.5 | 4.6 | 23.5 | 59 | 78.6 | 2.68 | No | Non- | 40 | 2.51 | SLE | Hematuria,  Bruises | Etamsylate | 7 |  |
| 41 | M | 2.5 | 22.4 | 77.5 | 3.63 | 18.8 | 3.2 | 2.7 | 1.6 | 1.2 | 10.7 | 68.7 | 87.2 | 3.63 | No | Non- | 201 | 2.29 | MP | Epistaxis | FFP | 4 |  |
| 42 | F | 2.75 | 16.5 | 68.2 | 3.28 | 19.7 | 5.1 | 3.1 | 2.6 | 2.4 | 24.1 | 89.2 | 69.2 | 3.28 | No | Non- | 187 | 1.42 | MP＋Flu A | A | Untreated | 0 |  |
| 43 | F | 7.75 | 16.3 | 65.9 | 2.78 | 20.1 | 14.9 | 9.8 | 15.4 | 5.1 | 23.8 | 89.3 | 68.3 | 2.78 | No | Non- | 113 | 1.73 | SLE | Epistaxis,  Hematuria,  Hemorrhagic spots | RBC,  VitK | 7 |  |
| 44 | M | 4.08 | 15.9 | 67.3 | 2.95 | 19.4 | 7.6 | 2 | 3.7 | 3.4 | 24.9 | 79.3 | 80.4 | 2.95 | No | Non- | 118 | 1.8 | MP | Hematemesis | FFP | 4 |  |
| 45 | M | 1.17 | 14.8 | 59.6 | 2.15 | 19.8 | 11.3 | 13.6 | 16.8 | 7.1 | 29.5 | 77 | 60.7 | 2.15 | No | Non- | 383 | 1.64 | Flu A | Lip trauma | Suture | 3 |  |
| 46 | F | 4.08 | 16.3 | 86.5 | 2.79 | 18.4 | 21.2 | 20.3 | 24 | 6.5 | 30.5 | 103.6 | 68.4 | 2.79 | No | Non- | 368 | 2.32 | ADV | A | Untreated | 0 |  |
| 47 | F | 4 | 14.6 | 89.2 | 2.09 | 20.5 | 11.6 | 5.6 | 5.1 | 2.5 | 29.8 | 89.3 | 80.4 | 2.09 | No | Non- | 454 | 2.35 | SARS-CoV-2 | Epistaxis | Consultation | 2 |  |
| 48 | M | 7.42 | 19 | 64.9 | 3.62 | 19.5 | 10.4 | 6.8 | 4.1 | 3.2 | 18.8 | 78.2 | 69.5 | 3.62 | No | Non- | 134 | 1.45 | MP＋Flu A | A | Untreated | 0 |  |
| 49 | F | 7.33 | 16.6 | 40.4 | 1.75 | 21 | 23.5 | 13.2 | 12.7 | 11.6 | 27.4 | 89.3 | 86.4 | 1.75 | No | Non- | 283 | 1.50 | SLE | A | Untreated | 0 |  |
| 50 | M | 3.92 | 22.6 | 75.1 | 1.98 | 21.4 | 15.7 | 11.5 | 12.6 | 9.3 | 17.3 | 89.2 | 83.5 | 1.98 | No | Non- | 271 | 1.63 | MP | Hemorrhagic spots | Consultation | 2 |  |
| 51 | F | 4.33 | 15.7 | 108.3 | 3.09 | 18.9 | 8.2 | 5 | 3 | 2.6 | 29.9 | 88.7 | 88.2 | 3.09 | No | Non- | 146 | 1.83 | MP | Epistaxis | VitK | 4 |  |
| 52 | M | 9.42 | 14.8 | 101.8 | 3.45 | 19.7 | 2.9 | 2.7 | 2.8 | 3.6 | 32.1 | 95.7 | 70.3 | 3.45 | No | Non- | 140 | 2.43 | Flu A | A | Untreated | 0 |  |
| 53 | M | 6.83 | 17.5 | 70.7 | 2.29 | 19.1 | 18.5 | 13.2 | 15.2 | 9.5 | 20.3 | 85.1 | 74.3 | 2.29 | No | Non- | 112 | 1.90 | ADV | A | Untreated | 0 |  |
| 54 | M | 5.5 | 16.9 | 42.5 | 1.86 | 21 | 30.1 | 18.4 | 17.3 | 15.2 | 20.5 | 90.4 | 87.5 | 1.86 | No | Non- | 180 | + | Flu A | Hemoptysis, Hematemesis, Hematochezia,  Hematuria,  Epistaxis | RBC,  Etamsylate,  Packing | 7 |  |
| 55 | F | 8 | 16.4 | 43.1 | 1.89 | 20.6 | 22.5 | 20.4 | 18.2 | 17.5 | 21.5 | 78.4 | 80.5 | 1.89 | No | Non- | 357 | 1.58 | Flu A | Hematemesis,  Epistaxis | Etamsylate,  Packing,  burning | 7 |  |
| 56 | F | 4.08 | 15.7 | 46.8 | 2.37 | 19.4 | 29.1 | 16.7 | 14.4 | 15.6 | 28.9 | 80.3 | 87.1 | 2.37 | No | Non- | 301 | 1.65 | SARS-CoV-2 | Hematemesis,  Epistaxis | Consultation | 4 |  |
| 57 | M | 11 | 16.2 | 41.9 | 2.58 | 18.8 | 28.4 | 16.1 | 13.7 | 11.3 | 28.1 | 95.3 | 80.3 | 2.58 | No | Non- | 277 | 1.48 | Flu A | Bruises | Consultation | 2 |  |
| 58 | M | 4.42 | 17.6 | 70.3 | 2.54 | 18.8 | 20.2 | 15.1 | 10.3 | 10.1 | 20.2 | 78.3 | 102.3 | 2.54 | No | Non- | 138 | + | SARS-CoV-2 | Epistaxis | Consultation | 2 |  |
| 59 | F | 5.33 | 16.6 | 112 | 1.8 | 19.5 | 10.3 | 7.2 | 7.9 | 4.6 | 23.9 | 85.3 | 85.5 | 1.8 | No | Non- | 248 | 2.29 | Flu A | Epistaxis | VitK | 4 |  |
| 60 | M | 7.92 | 17.6 | 80.3 | 2.15 | 18.3 | 21.0 | 18.4 | 16.7 | 13.9 | 26.2 | 104.2 | 98 | 2.15 | No | Non- | 284 | 2.16 | Flu A | Epistaxis | Consultation | 2 |  |
| 61 | F | 6.17 | 14.6 | 49.9 | 1.65 | 20.2 | 30.7 | 16.1 | 13.6 | 26.8 | 33.5 | 122 | 87.7 | 1.65 | No | Non- | 447 | + | ADV | Epistaxis | Etamsylate | 4 |  |
| 62 | F | 4.67 | 15.9 | 79.6 | 2.79 | 20.2 | 4.5 | 2.1 | 3.8 | 2.6 | 27.6 | 79.8 | 95.1 | 2.79 | No | Non- | 302 | 1.63 | Flu A | Epistaxis | Consultation | 2 |  |
| 63 | M | 7.17 | 15.2 | 64.8 | 2.98 | 19.8 | 18.5 | 16.3 | 15.9 | 11.8 | 27.2 | 89.3 | 94.3 | 2.98 | No | Non- | 364 | 2.07 | MP | A | Untreated | 0 |  |
| 64 | F | 11.67 | 14.5 | 44.7 | 2.41 | 18.9 | 10.3 | 8.9 | 5.1 | 4.2 | 30.2 | 87.2 | 90.3 | 2.41 | No | Non- | 46 | 1.88 | SLE | A | Untreated | 0 |  |
| 65 | F | 4.08 | 14.5 | 64.2 | 2.12 | 19.3 | 12.6 | 4.1 | 8.6 | 3.5 | 24.7 | 99.7 | 92.9 | 2.12 | No | Non- | 234 | 1.63 | Flu B | Epistaxis,  Bruises | PCC | 5 |  |
| 66 | F | 4.83 | 14.4 | 76.5 | 3.09 | 18 | 3.7 | 3 | 3.1 | 2.1 | 32 | 103.6 | 111.5 | 3.09 | No | Non- | 178 | 2.01 | SARS-CoV-2 | Epistaxis | Etamsylate | 4 |  |
| 67 | F | 7.83 | 15.5 | 50.2 | 2.57 | 18.2 | 16.4 | 10.4 | 8.5 | 5 | 25.1 | 80 | 84.5 | 2.57 | No | Non- | 198 | 1.65 | Flu A | Epistaxis | Consultation | 2 |  |
| 68 | M | 9.08 | 15.4 | 57.6 | 3.8 | 18.5 | 10.5 | 6.4 | 6 | 5.2 | 25.5 | 79 | 82.3 | 3.8 | No | Non- | 209 | 1.88 | MP | A | Untreated | 0 |  |
| 69 | M | 6.25 | 16.6 | 61.4 | 2.42 | 19.6 | 8.3 | 6.2 | 6.6 | 3.5 | 26.1 | 97.3 | 83 | 2.42 | No | Non- | 256 | 2.15 | Unknown cause | Epistaxis | PCC,  Etamsylate | 4 |  |
| 70 | M | 11.92 | 14.6 | 51 | 2.48 | 19.2 | 27.9 | 18.2 | 18.5 | 16.3 | 32.8 | 89.4 | 90.1 | 2.48 | No | Non- | 219 | 1.68 | Unknown cause | CVST | Anti-coagulaant therapy | 0 |  |

Abbreviations:Y, years; PT, prothrombin time; APTT, activated partial thromboplastin time; TT, thrombin time; Fg:C, fibrinogen activity; M, male; F, female; First diagnosis, subjects were first identified during coagulation screening or bleeding; No, uncorrected; A, asymptomatic; Clinical symptoms, Historical symptoms of bleeding and thrombosis were determined by patient interview and inspection of clinical records; PCC, prothrombin complex concentrate infusion; VitK, Vitamin K infusion; RBC, Red blood cell suspension infusion; FFP, Fresh Frozen Plasma transfusion; Untreated, without any coagulation factors replacement therapy; BAT bleeding scores, International Society on Thrombosis and Haemostasis bleeding assessment tool; ND, not detect; Non-, nonparallelism; CVST, cerebral venous sinus thrombosis; a, MDA showed there was a nonparallelism of endogenous coagulation factors activity, that is, the decreased levels of (FVIII:C, FIX:C, FXI:C, FXII:C) increased with the increase of dilution ratio and returned to normal levels; ^b^, virus infection without documented pathogen; ^c^, coagulation factors replacement therapy for control or prevention of bleeding.
